# Diabetes Mellitus

**Published:** 1845-08-01

**Authors:** 


### Diabetes Mellitus.

Communicated for the Buffalo Medical Journal.

About six years ago, some observations of mine on Diabetes Mellitus, were published in the "Boston Medical and Surgical Journal," under the caption of a "Medical Question"; and were in effect, "whether Diabetes Mellitus might not be symptomatic of disordered Brain?"

In forty years I have seen several cases of Diabetes; some of them have been under my personal care; I have had, also, opportunities to make Post Mortem examinations of some who have died of this disease. I was not aware when the article was published in the "Journal," nor do I now know, that the suggestion had been made, or the opinion entertained, that a disordered Brain might be the cause, or among the causes of Diabetes. The infrequency of its recurrence necessarily detracts from the value of opinions deduced from individual observation and experience. It was hoped, therefore, that the remarks in the "Journal," would attract some notice, inasmuch as Diabetes, considered as a distinct disease, has proved most intractable and fatal. The "Medical Question," however, remains unanswered; and for aught I know, unnoticed—"It fell still-born."

In September, 1842, the late Dr. Ives* of Suffield, Conn. desired me to see his townsman, Mr. Rogers, who had Diabetes.

Mr. Rogers was a farmer, 44 years old, regular in all his habits of living; and had been heretofore a healthy and laborious man. "A staunch New England farmer, who cultivated his own fields, and they yielded a bountiful return." It is not necessary to describe in detail the condition of Mr. R. at that time. It is enough to say here, that he had undoubted and confirmed Diabetes Mellitus; —the symptoms are familiar to medical men.

On explaining my views on the subject of Diabetes to Dr. Ives, he promptly consented to treat Mr. R. with reference mainly to the condition of the Brain. It was some time, perhaps three months after Mr. R. was under treatment, before there was any decided improvement; but from that time he gradually recovered; and I have the pleasure to know that Mr. R. has now comfortable health; and if he is not so stout and enduring as formerly, he is able to superintend and take an active part in the labors of his farm. He has entire immunity from Diabetes, except that strong mental (or Cerebral) excitement from whatever cause, is sure to be followed by discharges of honied urine. Mr. R., notwithstanding, considers himself a pretty sound and healthy man.

I am happy to be able to subjoin a brief history of Mr. R. from his own dictation—three years, now, since the commencement of his indisposition in June, 1842.

"I was brought up to labor on a farm. When I was 16 years old I had an inflammation of the thigh, that resulted in Caries of the bone. This gave me much trouble for several years; often inflaming, suppurating and discharging fragments of bone. In June, 1842, my thigh, which had been entirely healed for eight years, again inflamed, and I was apprehensive

* The profession and public have suffered a great loss in the death of Dr. Ives. He died of congestive fever, after a few days illness, in the middle of life and usefulness, December, 1844.

it was about to break out anew. But the application of poultices dissipated the inflammation, and I had no farther trouble from it. I had been subject to frequent and profuse "nose-bleed" from early life, especially in warm weather, but for some months before the time I now date the commencement of my illness, (June 1842,) I had none of it; and excepting a few turns of it in July following, I had none, nor has it recurred so frequently since as formerly. In June, 1832, I was preparing for the heavy work of summer, haying, &c., and though I labored hard daily, and my appetite was never better, I was, nevertheless, unhappy; my hired men provoked me, and we quarrelled—there was not (so I thought) the usual care and order in my domestic concerns, and I found much fault. I cannot describe my ill-feelings, and I had no assignable cause for them; I was easy and prosperous in my circumstances, and had always been happy in my domestic relations. So went on the months of July and August. My appetite was great, and my thirst inordinate. I often drank in such quantities that the stomach rejected it. In all this time I continued to labor, though it was obvious to myself that I was losing flesh and strength daily. I tired soon, my skin was harsh and dry, (very unusual) and my bowels were constipated, seldom moving oftener than once in five or six days. It was not until September that I became aware of passing an unusual quantity of urine, and my attention was particularly attracted by a singular deposit where some drops fell on my dress. I examined this and found it to be sweet as sugar; this surprised me, and yielding to the remonstrances of my family, I consulted Dr. Ives. It was past the middle of September when I came under the care of Dr. Ives and yourself. I am now, (after three years) as fleshy as I ever was, and as able to labor as most farmers of my age. If I am vexed or perplexed and have a sleepless night or two, I am certain to have discharges of honied urine, with great thirst. But a hot salt water bath and a cathartic restores me; so that I seldom trouble my friends or the doctor. You will allow me to add that the greatest harmony prevails in my family, and my hired men find no fault with me."

Many, if not most of the leading symptoms in Diabetes Mellitus, can be accounted for in no other way than on the supposition of a disordered Brain. It may be ascertained too, always, probably, that for a longer or shorter time previous to the occurrence or development of diabetic symptoms, there has existed evidence of disordered functions of the Brain; and afterward, in the progress of the disease, it will be observed that the patient becomes morally obtuse, stolid, fatuous, and at length, dies comatose, apoplectic; and finally, that Post Mortem examination discloses no sufficient cause of death, except it be the unnatural condition of the Brain.

In 1843, Mr. S., aged 25 years, after languishing a year and a half, died of Diabetes Mellitus. The circnmstances of the case were briefly these. Mr. S. left the labors of a farm in Massachusetts, and was employed two years as an assistant teacher in an Academy in Virginia. On his return from Virginia he resumed his farming; it was not long, however, after his return, before it was apparent that he had undergone a great moral change. Instead of the mild, good-natured, and retiring young man that he *was*—he had become irritable, irascible, boisterous, and peremptory. "It seems," said his good mother to me, "as though William was crazy." It was after this time, and not until it was discovered that he passed an unusual quantity of urine, that he came under my notice and care. My opportunities of seeing Mr. S. daily, as it were, from the time he came under my care, together with the P. Mortem examination, (which will be given below,) led to the views I entertain of the cause of Diabetes Mellitus, as I have succinctly stated them in introducing this case.

The Post Mortem examination of Mr. S. was made with care, and in presence of several medical gentlemen who witnessed what I am about to state. The body was greatly emaciated, a picture of famine and attenuation, and quite bloodless. The organs of the Pelvis, Abdomen, and Chest except a few open tubercles in the apex of one lung, apparently of recent origin, were sound; they had undergone no visible structural change. The Brain, on free exposure, showed at sight, no trace of previous or present disease. The membranes were remarkably white, pellucid and dry. On separating the lobes, the ventricles, also, were found empty and dry. The Brain itself—the mass, resembled its model in Plaster; it was white, firm, hard, and solid, to a degree we had never witnessed before; when cut into, it reminded us of the strumous mamma and testes; and the resemblance was so striking, that the question arose then, whether the change in the substance of the Brain might not have arisen from a similar cause or causes, (be they what they may) that produce analogous changes in the glands referred to.

I take occasion to remark here, that some weeks before the death of Mr. S., when he had become very stupid and senseless, and finally comatose, his urine diminished to much less than the natural quantity, and instead of the sweet and honied taste, it was pungently acid; like drops of Sulphuric Acid in water.

Circumstances antecedent and present, determined our treatment of Mr. Rogers. It consisted in general blood-letting, and topical about the head; and by puncturing the septum now and then, we succeeded, measurably, in restoring the habitual hemorrhage from the nose. He took small doses of calomel and opium at intervals, for some time, interposing cathartics as they were required. The capillary circulation was promoted by general baths of salted water, at, or above 100°; foot-baths with mustard, blisters, &c., &c. He was enjoined a regular diet, though a particular or select one was not insisted on. It consisted of plain, substantial animal and farinaceous food, morning and noon, interdicting all food and drink except at the prescribed periods. After eating, his drink was *hot* tea—ad libitum.

Mr. R. was a man of sense, of sound mind and firm of purpose, and when he was made acquainted with our views of his case, and with our proposed plan of treatment, he submitted himself unreservedly; and by scrupulously guarding against exciting causes, and manfully resisting his sickly craving, he contributed, no doubt, essentially to his cure. F.

---

The subject of the foregoing communication possesses much pathological interest and practical importance. Modern Pathology has advanced our knowledge of Diabetes vastly, since the time, within the recollection of those of us who are still junior members of the profession, when it was regarded as a renal disease. Recent researches, which have led to a better knowledge of the changes which the alimentary supplies undergo during the process of assimilation, and of the function of secretion, have enabled us to perceive that the presence of Sugar in the urine of diabetic patients, is the result of a deficiency of that *converting* power, (to quote the term used by Dr. Prout,) by which Sugar, amylaceous and other substances of the aliment, or the separated portions of the organism, are changed into organic products, fitted for the nutritive and other normal functions. Owing to this deficiency of converting power, the Sugar contained in the aliment enters the blood vessels unchanged, and the farina, and other substances, obeying their chemical tendencies, pass into the form of saccharine matter. The Sugar thus exists in the blood, and the only part which the kidneys perform, is to separate it from this fluid. The disease, therefore, has been ascertained to consist (in so far as the development of sugar is concerned) in mal-assimilation, not in mal-secretion. We have thus made great progress in the investigation of this disease ; but we have only reached a secondary stage in the investigation. The true proximate cause of the disease is ulterior to the introduction and formation of sugar in the blood. Whence is derived the absence of the normal converting power? This question remains to be answered. It is with reference to this question that the views presented in the foregoing communication have especial interest. May it not be, that this deficiency of converting power, or in other words, this mal-assimilation, to which our investigations have carried us, is, in fact, the consequence of a pre-existing affection of the nervous system? The affirmative to this inquiry is invested with much plausibility from the fact, that in the present state of our knowledge, we must attribute the prime source of all the vital functions to the *vis nervosa* — the assimilatory among the rest. The two cases which our correspondent details are highly interesting and important in this connection. That the disease stands in certain close relations with a disordered condition of the nervous system, these, and other cases, sufficiently attest. But, whether in these relations the diabetic affection be first or second in the order of sequence, or whether both are effects of the same, unknown, ulterior, pathological condition, remains to be definitively settled by future researches. The subject commends itself to, and we trust will receive the attention of practical observers and pathologists.

Our correspondent, who chooses to withold his name, promises to contribute other papers, as his leisure and inclination allow, on topics which may present themselves on a retrospection of forty years of active professional experience. To express, in behalf of the Journal, our sense of the value of such communications, would be superfluous. It were much to be desired, that practical members of our profession, after long experience, would publish oftener than they do, the important facts, and the prominent views which appertain to their observations and reflections; and we shall be truly glad if our Journal may contribute to any extent to promote this desirable object. An Autobiography of a Physician of forty or more years of active service, which would embrace an account of the anxieties, difficulties, mortifications, temptations, and peculiar trials pertaining to his medical career, as well as the results of his observations and meditations on medical topics; and more than all, which would communicate an honest confession of his false steps, and blunders, as well as his success, would be one of the most entertaining and instructive books which could be written. Whoever, possessing the proper qualifications, will write such a book, will add to the useful labors of the most useful of callings, a legacy of great value to those who are following, and are to follow in his footsteps.—Editor.

